# Redundant-target processing is robust against changes to task load

**DOI:** 10.1186/s41235-017-0088-x

**Published:** 2018-02-21

**Authors:** Stephanie A. Morey, Nicole A. Thomas, Jason S. McCarley

**Affiliations:** 1 College of Education, Psychology and Social Work, Flinders University, Adelaide, SA Australia; 2 College of Healthcare Sciences, James Cook University, Cairns, QLD Australia; 3 School of Psychological Science, Oregon State University, Corvallis, OR USA

**Keywords:** Capacity coefficient, Limited capacity, Multi-task, Redundancy gain, Redundant signals effect, Super-capacity, Target detection, Workload capacity, Workload resilience

## Abstract

Monitoring visual displays while performing other tasks is commonplace in many operational environments. Although dividing attention between tasks can impair monitoring accuracy and response times, it is unclear whether it also reduces processing efficiency for visual targets. Thus, the current three experiments examined the effects of dual-tasking on target processing in the visual periphery. A total of 120 undergraduate students performed a redundant-target task either by itself (Experiment 1a) or in conjunction with a manual tracking task (Experiments 1b–3). Target processing efficiency was assessed using measures of workload resilience. Processing of redundant targets in Experiments 1–2 was less efficient than predicted by a standard parallel race model, giving evidence for limited-capacity, parallel processing. However, when stimulus characteristics forced participants to process targets in serial (Experiment 3), processing efficiency became super-capacity. Across the three experiments, dual-tasking had no effect on target processing efficiency. Results suggest that a central task slows target detection in the display periphery, but does not change the efficiency with which multiple concurrent targets are processed.

## Significance statement

High-workload environments often mean dividing attention between multiple visual tasks or displays. The current study examined aspects of visual display design that might influence target detection in multi-task environments. Using paired target discrimination/manual tracking tasks, we investigated the effects of target redundancy on participants’ ability to notice eccentric visual signals while engaged in a central task. Our goal was to assist display design by identifying factors that help multi-tasking operators to notice visual alerts and alarms in their peripheral vision.

## Background

Operators in high-stress domains often need to divide attention between the central and peripheral visual fields. A pilot, for example, must also monitor for cockpit alerts while maintaining awareness of an aircraft’s position in space (Wickens, Sebok, McCormick, & Walters, 2016), and operators in air traffic control must remain responsive to critical alerts while managing the flow of air traffic (Imbert et al., 2014). Similarly, the increasing use of head-worn displays in professional roles means that many operators are required to switch attention between tasks within their central visual field and peripheral events projected onto the headset (Pascale et al., 2015). Within each of these domains, performing effectively means processing information presented centrally, while also discriminating between critical and non-critical “noise” events in the visual periphery. For system designers, this issue implies a need to understand the task and display characteristics that maximize peripheral detection and discrimination under conditions of high central load.

An obvious technique to improve target detection is to increase target salience, the feature contrast between the target and its surroundings (Itti & Koch, 2000; Theeuwes, 2010). Unfortunately, visual heterogeneity reduces feature contrast (Humphreys, Quinlan, & Riddoch, 1989; Nothdurft, 1992), and in a cluttered, dynamic environment like the cockpit, even events designed to be highly salient can go undetected (Nikolic, Orr, & Sarter, 2004; Steelman, McCarley, & Wickens, 2013). Alternative strategies for ensuring rapid target detection are, therefore, useful. One converging strategy is to present targets redundantly, that is, on multiple channels simultaneously. Redundant presentation generally speeds target detection (Miller, 1982; Todd, 1912), and is endorsed in human factors engineering as a method of promoting information security (Wickens & Hollands, 2000; Wickens, Prinet, Hutchins, Sarter, & Sebok, 2011). For example, vehicle collision warning systems often employ redundant visual or auditory signals to alert a driver of a potential collision (Ho, Reed, & Spence, 2007). Similarly, in aircraft settings, pilots respond faster to missile approach warnings as the number of informational channels delivering the warning increases (Selcon, Taylor, & McKenna, 1995).

Like a manipulation of salience, however, redundant information display is not guaranteed to aid performance. Constraints on processing resources can modulate the efficiency with which concurrent events are processed (Townsend & Eidels, 2011), limiting the benefits produced by a redundant target (e.g., Eidels, Townsend, Hughes, & Perry, 2014; McCarley, Mounts, & Kramer, 2007; Townsend & Nozawa, 1995). Moreover, under some conditions, the addition of the second target may produce no redundancy gain at all (Grice, Canham, & Gwynne, 1984). More surprisingly, within a multi-task environment redundant signals may actually be disruptive: Wickens and colleagues (Seagull, Wickens, & Loeb, 2001; Wickens & Gosney, 2003) have reported evidence that redundant audio-visual target presentation in a monitoring task can disrupt performance in an ongoing tracking task. These results suggest that the demands of encoding or recognizing redundant targets can divert processing resources from a concurrent task, producing interference. In the current experiments, we pursue this effect by examining the converse possibility, that the demands of a concurrent central task might limit the efficiency of redundant signal processing.

### Measuring the efficiency of redundant-target processing

In a standard redundant-target task, participants make a speeded response to a target presented in either of two channels (e.g., on a visual channel and an auditory channel). On single-target trials, a target appears in only one channel (e.g., only the visual channel); on redundant-target trials, the target is presented in both channels (e.g., on both the visual and auditory channels). The observer responds as soon as a target is detected in either channel, a condition known as a *first*-*terminating* stopping rule (Colonius & Vorberg, 1994). Under these conditions, redundant signals generally produce faster responses than single targets, a phenomenon known as a *redundant signals effect* (RSE) or *redundancy gain* (Miller, 1982). For example, for a driver approaching a railway crossing, the presentation of both a red flashing light and a loud bell is likely to allow faster detection, and consequently a faster braking response, than either warning presented alone.

The RSE, however, may differ in magnitude under different task constraints, and in some cases, may be entirely absent. The size of the RSE reflects variations in a cognitive system’s *architecture* and *workload capacity* (Townsend & Eidels, 2011; Townsend & Nozawa, 1995), where architecture refers to the arrangement of channels (e.g., serial or parallel), and workload capacity refers to the efficiency with which the channels operate concurrently. In addition, the RSE can also reflect variations in inter-channel dependencies (Townsend & Wenger, 2004). The simplest model of the RSE is the *unlimited*-*capacity, independent parallel* (*UCIP*) model, wherein multiple channels operate with stochastic independence and each channel’s rate of processing remains unchanged, regardless of the total number of channels under operation (Townsend & Eidels, 2011). Under a first-terminating stopping rule, the UCIP model produces a redundancy gain simply because the processing time of the system as a whole is based on the output of the fastest channel on each trial. This mechanism is known as *statistical facilitation* (Raab, 1962). *Super*-*capacity* occurs when an increase in the number of operating channels (i.e., workload) results in a corresponding increase in the individual channels’ processing rates, producing a larger RSE than predicted by the UCIP model. Conversely, *limited capacity* exists when an increase in workload decreases the processing rates of the individual channels, producing a smaller RSE than predicted by the UCIP model. In situations where capacity is highly limited, the redundancy gain may be no different to that of a serial model.

Importantly, unless capacity is extremely limited, mean response times (RTs) alone cannot distinguish gradations in parallel processing capacity within a redundant-target task. To establish whether a system is limited, unlimited, or super-capacity, we therefore need to analyze the data at the level of the RT distributions. As a means of distinguishing between statistical facilitation in the UCIP model and actual processing speed-ups with multiple channels, Miller (1982) established an upper bound on performance for the UCIP model, known as the *race*-*model inequality*. The inequality holds that in the UCIP model, the cumulative distribution function (CDF) of the redundant-target trials cannot exceed the combined CDFs for the two categories of single-target trials. Evidence that the CDF for the redundant-target trials exceeds the summed CDFs for the single-target trials at any time *t* thus disconfirms the UCIP model and implicates a super-capacity model instead. Analogously, Grice et al. (1984) identified a lower bound on UCIP performance, providing a test of extreme capacity limitations. The Miller and Grice inequalities, however, are both conservative tests that are insensitive to modest variations in capacity. Townsend and Nozawa’s (1995) workload capacity coefficient, *C*(*t*), provides a more fine-grained measure of efficiency, sensitive to variations in between the Miller and Grice boundaries.

*C*(*t*) rests on the conceptualization of the hazard function for speeded responses as a gauge of moment-to-moment cognitive expenditure. In a speeded task, the hazard function, *h*(*t*), indicates the instantaneous probability with which a response will occur at time *t*, given that a response has not yet occurred (Townsend & Ashby, 1983). The integrated hazard function, *H*(*t*), is the integral of the hazard function up to time *t*. Importantly, within the UCIP model, the integrated hazard functions for multiple operating channels are additive. In other words, if processing follows the UCIP model, the value of the integrated hazard function in the redundant-target condition at time *t* is equal to the sum of the values of the integrated hazard functions of the two single-target conditions at time *t*. Taking advantage of this constraint, Townsend and Nozawa (1995) define the capacity coefficient, *C*(*t*) as,

1 $$ C(t)=\frac{H_{AB}(t)}{H_A(t)+{H}_B(t)},\kern0.5em t>0, $$

where *H*_AB_(*t*) refers to the integrated hazard function of the redundant-target condition, and where *H*_A_(*t*) and *H*_B_(*t*) refer to the individual integrated hazard functions for a target present only on channel A or channel B, respectively. Under the UCIP model, in which the integrated hazard functions for channels A and B are additive, *C*(*t*) = 1.0. Values of C(*t*) greater than 1.0 indicate that *H*_AB_(*t*) > *H*_A_(*t*) + *H*_B_(*t*), implying super-capacity. Conversely, values less than 1.0 indicate that *H*_AB_(*t*) < *H*_A_(*t*) + *H*_B_(*t*), implying limited capacity. In extreme cases capacity may be fixed, *C*(*t*) = 0.5, implying a zero-sum tradeoff between channels and producing performance akin to that predicted by a serial model.

A transformation of *C*(*t*) that can be used to compare performance across experiments is the standardized capacity score, *Cz* (Houpt & Townsend, 2012). *Cz* provides a summary capacity measure collapsed over time and suitable for comparison between experimental conditions. Values follow a standard normal distribution, with a score of 0 indicating UCIP-level processing, positive scores indicating super-capacity, and negative scores indicating limited capacity.

The capacity coefficient was developed for examining judgments of displays wherein, on single-target trials, the position of the potential second target is empty. Recent developments have extended the approach to accommodate analysis of displays in which single-target conditions include a distractor in place of the empty space (Little, Eidels, Fific, & Wang, 2015). The measure of processing efficiency in this case has been termed *resilience*, *R*(*t*) (Little et al., 2015). *R*(*t*) is calculated with the formula used to calculate *C*(*t*), except that the integrated hazard functions in the denominator of the equation represent single-target conditions on which a distractor is present,

2 $$ R(t)=\frac{H_{AB}(t)}{H_{AX}(t)+{H}_{XB}(t)},\kern0.5em t>0, $$

where *H*_*AX*_(*t*) is the integrated hazard function for single target A accompanied by a distractor, *X*, and *H*_*XB*_(*t*) is the integrated hazard function for single target B accompanied by the *X. R*(*t*) can, in turn, be converted to a measure of normalized resilience (Houpt & Little, 2017), referred to here as *Rz*, analogous to *Cz*. Resilience differs from capacity because, when a distractor is present on single-target trials, it can divert processing resources from the target, slowing target detection (Allen, Madden, Groth, & Crozier, 1992; Ben-David, Eidels, & Donkin, 2014). Resilience, therefore, reflects both the changes in target processing rate that occur as the number of targets increases, and the potential release from interference that occurs when a distractor is replaced by a target.

Interpretation of resilience scores is more involved than interpretation of the workload capacity scores. By definition, channels in the UCIP system operate at the same rate regardless of processing load. Thus, the UCIP model predicts a benchmark value of *R*(*t*) = 1 (*Rz* = 0), just as it predicts a benchmark value of *C*(*t*) = 1 (*Cz* = 0). More generally, a parallel self-terminating model predicts that *R*(*t*) will not vary as a function of distractor discriminability, and that redundant-target processing will be equally efficient in the experimental designs with and without distractors, that is, *C*(*t*) and *R*(*t*) will be equal (Little et al., 2015).

In contrast, a serial self-terminating (SST) model predicts that *R*(*t*) will vary with the relative discriminability of the target and distractor. For simplicity, assume a case in which the integrated hazard functions for targets A and B are identical, both with distractors (*H*_*AX*_(*t*) = *H*_*XB*_(*t*)), and without (*H*_*A*_(*t*) = *H*_*B*_(*t*)). On redundant-target trials, the first item processed will always be a target. The integrated hazard function for redundant-target trials will, therefore, equal the integrated hazard function for single-target trials without distractors, i.e., *H*_*AB*_(*t*) = *H*_*A*_(*t*). This reduces Eq. 2 to,

3 $$ R(t)=\frac{H_A(t)}{2\times {H}_{AX}(t)},\kern0.5em t>0. $$

On single-target trials, assuming the target position is unpredictable, the number of items that are processed will vary randomly from trial to trial; on some trials only the target will be processed, and on the remaining trials, the distractor will be processed before the target. The difference between *H*_*AX*_(*t*) and *H*_*A*_(*t*) will thus reflect the time needed to process the distractor on those trials on which the target is not processed first. When the time needed to process the distractor is negligible relative to the time needed to process the target, *H*_*AX*_(*t*) will equal *H*_*A*_(*t*), and *R*(*t*) will be fixed. When the time needed to process the distractor becomes more substantial, *H*_*AX*_(*t*) decreases and *R*(*t*) becomes larger. In other words, the SST model predicts that resilience will be limited when distractor interference is negligible and will increase as distractor interference becomes larger.

But regardless of the underlying architecture, values of *R*(*t*) < 1 or *Rz* < 0 imply that redundant targets are processed slower than predicted by the UCIP model, and values of *R*(*t*) > 1 or *Rz* > 0 imply that redundant targets are processed faster than predicted by the UCIP model (Houpt & Little, 2017). By analogy to the terminology applied to workload capacity, we will describe these effects as limited capacity and super-capacity, respectively. However, it is important to note that these labels describe performance of the multi-channel system as a whole and do not necessarily connote changes in the processing rates of the individual channels. As described above, for example, changes in distractor discriminability within an SST system may change *R*(*t*) from less than 1 to greater than 1, even if the target processing rate remains constant.

Redundant presentation of peripheral signals will thus aid detection only if the signals are processed with spare capacity or resilience. Unfortunately, existing data do not make it clear that this will be the case. Empirical data suggest that attention is weighted toward the central visual field (Carrasco, Evert, Chang, & Katz, 1995; Carrasco & Yeshurun, 1998; Wolfe, 1998), and modeling likewise suggests that elemental processing resources are denser in the central retina than in the eccentricity (Miller & Ulrich, 2003). A demanding task in the central visual field might further shift attention away from the retinal eccentricity (Leibowitz & Appelle, 1969; Reimer, 2010), engendering *visual tunneling* (Williams, 1985). For example, observers have higher detection thresholds for luminance probes in the visual periphery when performing a concurrent central task, with more difficult central tasks producing larger threshold increases (Leibowitz & Appelle, 1969). Similarly, accuracy on a peripheral discrimination task is higher when a concurrent central task is low in perceptual load than when it is high (Williams, 1985). Even task-irrelevant stimuli presented at fixation can interfere with processing of peripheral visual targets (Beck & Lavie, 2005; Schwartz et al., 2005). Within a peripheral redundant-target paradigm with a simultaneous central-load task, such effects might limit processing resilience of peripheral targets, reducing the magnitude of the RSE. In addition, a prominent account of dual-task performance, multiple resource theory, argues that resource competition between tasks drawing on similar processing resources will decrease performance (Wickens, 1981, 2002). According to this theory, within a dual tracking/target detection paradigm, the central tracking task may consume visual processing resources, limiting the attentional resources necessary for processing peripheral items. Based on such an effect, we would expect to see poorer efficiency when the detection task is accompanied by the central tracking task.

To test these possibilities, the current experiments assessed human performance within a dual-task paradigm pairing a central manual tracking task with a peripheral redundant-target task. We examined whether the detection of visual targets observed within a dual-task paradigm produces a redundancy gain, and if so, just how efficiently the processing compares to that of the UCIP model. In Experiments 1 and 2, we used a target detection task to assess processing resilience while performing under both single- and dual-task load. Finally, in Experiment 3, we designed stimuli to preclude parallel target processing to examine resilience within a serial model.

## General method

Here, we describe methods of stimuli and procedure common to all of the experiments that follow.

### Apparatus and stimuli

Stimuli were presented on a 27 Samsung LED monitor, with a resolution of 1920 × 1080 pixels (1 pixel was equal to 0.33 mm) and a refresh rate of 100 Hz. Participants completed the experiment at a viewing distance of approximately 600 mm, although viewing distance was not fixed. The experimental program was created using Presentation software Version 16.5 Build 09.17.13 (Neurobehavioral Systems, 2017). Tracking task performance and responses to the concurrent target detection task were collected via a Logitech Attack 3 (Logitech, 2017) joystick.

Stimuli for the target detection task were black capitalized letters, with Ts as targets and Ls as distractors. Letters appeared in the upper left (location A) and right (location B) of the screen with polar coordinates *θ* = ± 51.15° from the vertical midline and *r* = 21.79° of visual angle from the screen center point. The peripheral target/distractor stimuli were chosen randomly and with equal probability from among four combinations: redundant targets (TT), single target on left (TL), single target on right (LT), and redundant distractors (LL).

Peripheral stimuli appeared approximately 20 times per 60-s trial, and remained visible each time until the participant issued a joystick response or a timeout duration of 2000 ms was reached. To ensure that participants were unable to predict times at which a new target or distractor might appear in the periphery, the inter-stimulus interval between successive events in the peripheral channels was drawn from a delayed exponential distribution. The delayed exponential is the sum of a fixed delay value with a random value drawn from an exponential distribution. Here, the fixed delay was set to 1000 ms and the mean of the exponential component was set to 2000 ms. Because the exponential component of the distribution was memoryless, these parameters ensured that the interval between successive stimuli was at least 1000 ms, but was unpredictable beyond that.

**Fig. 1:**
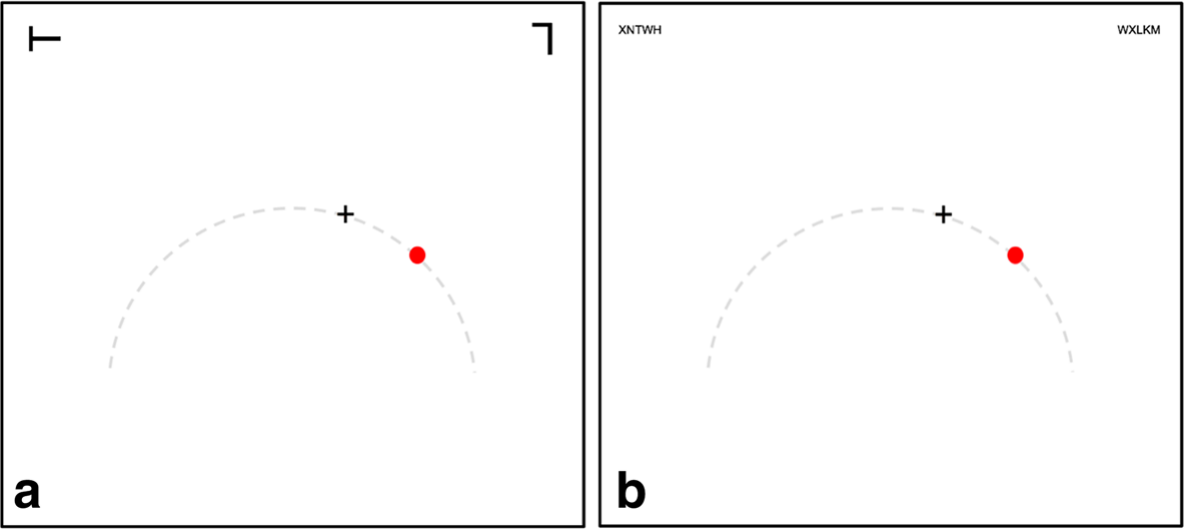
**a** A single-target dual-task trial from either Experiment 1b or the dual-task condition of Experiment 2. **b** A single-target dual-task trial from Experiment 3. The participant pressed a button when they detected the target (in the top left of these figures). The tracking task involved manually maneuvering the black cursor (+) with the moving red circle. The black cursor and the red circle moved along an invisible arc (presented here as a dashed line). Stimuli for the single- and dual-task experiments/conditions were similar, except the black cursor was not visible in the single-task versions

**Fig. 2:**
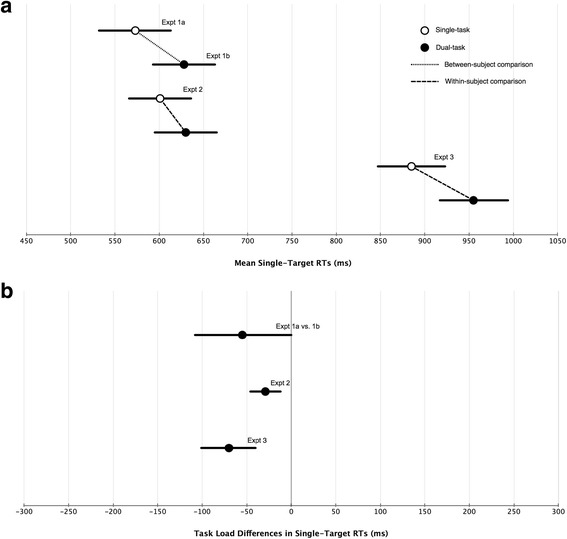
**a** Means and 95% BCIs for single-target RTs (ms) in each experiment. **b** Means and 95% BCIs on the task-load difference scores for single-target RTs in each experiment (single-task RT minus dual-task RT)

Stimuli for the pursuit tracking task were a black cursor “+” in size 10 Arial font (0.76° × 0.76° of visual angle) and a red circular marker (subtending 0.95°). Both the cursor and the marker moved along a semicircle, extending into the upper visual field, with a diameter of 19.85° centered along the horizontal midline of the display (see Fig. 2). The pattern of target motion was created by summing sinusoids with frequencies of 0.07, 0.15, and 0.23 Hz (Strayer & Johnston, 2001). The center point of the arc was 5.72° below the screen’s center point. The component sinusoids were randomly phase-shifted to produce a different pattern of motion on each trial. The cursor moved along the same arc, at a maximum rate of 80° per second, but required manual control via the joystick to maneuver. To increase task difficulty, at the start of each trial the red target appeared at a randomly selected location along the semicircular path, whereas the cursor always began centered along the path. Thus, only the red marker was visible to participants. In all the experiments, the coordinates of both stimuli were recorded every 100 ms (every three frames) throughout each of the 20 tracking intervals. Figure 1a presents a schematic stimulus representation from a left single-target dual-task trial from Experiment 1b or Experiment 2.

### Procedure

Participants completed the task in a well-lit room. At the start of the session, participants were instructed to hold the joystick with two hands, allowing both thumbs to rest on the buttons on top of the joystick. To perform the detection task, participants were instructed to remain aware of targets appearing in the upper regions of the screen. Participants were required to respond as fast as possible if a letter T appeared in either one or both peripheral stimulus locations, but to refrain from responding if both peripheral letters were Ls. Responses were made by pressing the buttons on top of the joystick with both thumbs. Bimanual joystick button responses ensured that both hemispheres were activated during the task. As our aim was to understand the attentional processes involved in target detection, using bimanual responses reduced the likelihood of any stimulus-response compatibility effects (e.g., congruency between targets and response hand).

To enhance engagement, both tasks were framed within a driving scenario in which participants were asked to imagine they were driving a vehicle to the university. For the tracking task, participants aligned the cursor, representing their car, with the red marker, representing an in-vehicle navigation system. For the target-detection task, participants were told to imagine that they were responding to traffic signals, where Ts represented red lights and Ls represented green lights. Thus, participants were required to brake by issuing a joystick button press as fast as possible if they encountered a red light (T), but to withhold responding if a pair of green lights (LL) appeared. Participants were encouraged to respond as fast as possible, while maintaining accuracy.

Each tracking interval lasted 60 s, after which participants were given the chance for a short break before starting the next interval. To begin a new interval, participants pulled the joystick trigger. Within each block, participants completed a total of one 60-s practice interval, followed by 20 experimental intervals (the number of blocks varied between experiments).

After finishing the experiment, participants completed the FLANDERS questionnaire. Participants were then asked if they held a current valid driver’s license, and if so, approximately how many years of driving experience they had. Finally, participants were debriefed and thanked for their time.

## Experiment 1a

Experiment 1a provided a baseline estimate of resilience for a parafoveal target detection task performed alone (i.e., in a single-task condition).

### Method

#### Participants

Twenty-five Flinders University undergraduate students (21 female; *M*_*Age*_ = 23.08 years, *SD* = 5.12, *Range* = 18–40) were recruited as part of a course requirement. All participants had normal or corrected-to-normal visual acuity and normal color vision, and were fluent in English. Participants were screened for right-hand dominance, with a minimum Flinders Handedness Survey (FLANDERS; Nicholls, Thomas, Loetscher, & Grimshaw, 2013) score of + 5 (*M* = + 9.76, *SD* = 0.66). Twenty participants held a current valid driver’s license, with between 0.5 and 15 years of driving experience (*M* = 4.69, *SD* = 3.61).

#### Apparatus and stimuli

In Experiment 1a, stimuli for the target detection task were a black capital T (target) and L (distractor) presented in 16-point Arial font (1.58° × 1.14° of visual angle) on a white background. Stimuli letters were randomly and independently rotated between 0° and 270°, in steps of 90°. In addition, the black cursor was invisible to ensure that participants did not attempt to perform the tracking task.

#### Procedure

In Experiment 1a, the participants’ only task was to monitor and respond to peripheral targets. As such, participants were instructed to ignore the movements of the red target circle and were not instructed to perform the tracking task. Participants completed one block of 20 60-s tracking intervals. The entire process took approximately 30 min.

#### Analysis

For statistical analysis, raw resilience scores, *R*(*t*), were converted to standardized resilience scores, *Rz*, (Houpt & Townsend, 2012) using the “sft” package (Houpt, Blaha, McIntire, Havig, & Townsend, 2014) for R (Core Team, 2016).

Analysis of RTs for correct responses, normalized resilience scores, and root mean squared error (RMSE) for tracking performance was performed through Bayesian parameter estimation using a Markov chain Monte Carlo (MCMC) sampling procedure (Kruschke, 2013, 2015; Lee & Wagenmakers, 2013). This approach begins by assuming a prior distribution on a parameter value of interest, then updates the prior through probabilistic sampling to approximate the posterior distribution on parameter values based on the observed data (Kruschke, 2015). Analyses were conducted using sampling functions from the JAGS package (Plummer, 2015) in R. RTs were analyzed in a one-way, within-participant design, with additive effects of condition (first single-target, second single-target, redundant targets) and participant. Effects were assumed to follow normal distributions with vague priors on their means and standard deviations. Following Kruschke (2015),

$$ {\displaystyle \begin{array}{l}{Y}_{\mathrm{participant}},{\kern0.5em }_{\mathrm{condition}}\sim N\left(a0+{a}_{\mathrm{participant}}+{a}_{\mathrm{condition}},\kern0.5em {\sigma_y}^2\right)\\ {}{\sigma}_y\sim U\left( SD/1000,\kern0.5em SD\ast 1000\right)\\ {}a0\sim N\left(M,\kern0.5em {\left[100\times SD\right]}^2\right)\\ {}{a}_{\mathrm{participant}}\sim N\left(0,\kern0.5em {\sigma_{\mathrm{participant}}}^2\right)\\ {}{a}_{\mathrm{condition}}\sim N\left(0,\kern0.5em {\sigma_{\mathrm{condition}}}^2\right)\\ {}{\sigma}_{\mathrm{participant}},\kern0.5em {\sigma}_{\mathrm{condition}}\sim \varGamma \left(\alpha, \kern0.5em \beta \right)\\ {}\alpha = SD/2\\ {}\beta =2\ast SD\end{array}} $$

where *Y*_participant, condition_ is the RT for a given participant in each condition, σ_*y*_ is the estimated standard deviation of the normal distribution of RTs, *a0* is the estimated grand mean RT, *a*_participant_ is the participant effect, *a*_condition_ is the condition effect, *M* is the grand mean of the observed RT scores, and *SD* is the standard deviation of the observed RT scores. Deflections from the grand mean representing effect of condition were constrained to sum to zero across conditions. Using the data sample mean and standard deviation to set parameters of the prior ensured that the prior distribution was scaled appropriately to the data (Kruschke, 2015). To test for the possibility of lateral (left versus right) attentional bias, along with redundancy gains, we estimated RTs in two different ways. In the first case, to check for the possibility of lateral asymmetries in performance, data were coded such that two single-target conditions represented the left single-target and right single-target trials. Thus, any difference in RTs in the first case would signal that participants had tended to respond to targets in one location faster than the other. In the second case, to provide a conservative estimate of the redundancy gain, data were coded such that the two single-target conditions represented the faster and slower mean single-target condition for each participant. Redundancy gain was defined as the difference between the shorter of the two single-target RTs and the redundant-target RT. This method of measuring redundancy gains provides more conservative estimates than the alternative approach of comparing redundant-target RT to the mean of the single-target RTs (cf. Biederman & Checkosky, 1970).

*Rz* and RMSE scores were estimated in a one-sample design (Kruschke, 2013),

$$ {\displaystyle \begin{array}{l}{Y}_{\mathrm{participant}}\sim N\left(u,\kern0.5em {\sigma}^2\right)\\ {}u\sim N\left(M,\kern0.5em {\left[100\times SD\right]}^2\right)\\ {}\sigma \sim U\left( SD/1000,\kern0.5em SD\ast 1000\right)\end{array}} $$

where *Y*_participant_ is the score for a given participant, *u* is the estimated grand mean score, σ is the estimated standard deviation of the normal distribution of scores, *M* is the grand mean of the observed scores, and *SD* is the standard deviation of the observed scores.

Each parameter estimate was based on four MCMC chains, run for 1000 burn-in steps, followed by 250,000 steps each. Chains were thinned to every fifth step in to reduce sample autocorrelation, leaving a total of 50,000 samples for analysis. All estimated parameters showed Gelman-Rubin statistic values (Gelman & Rubin, 1992) of 1.01 or less, indicating satisfactory convergence of the MCMC chains (Kruschke, 2015).

### Results

#### Error rates

Detection error rates were analyzed to ensure that participants had correctly followed instructions. As a general rule, the capacity coefficient is robust against error rates of up to 0.30 (Townsend & Wenger, 2004). No participants in Experiment 1a produced false alarm rates that exceeded this value (*M* = 0.10, *Range* = 0.01–0.20). Miss rates in all target conditions—single target on left (*M* = 0.01, *Range* = 0.00–0.09), single target on right (*M* = 0.01, *Range* = 0.00–0.07), and redundant targets (*M* < 0.01, *Range* = 0.00–0.07)—were very low. On average, participants correctly responded to approximately 72 trials in each condition: *M* = 71.80 (*Range* = 67–75) for left-targets, *M* = 71.68 (*Range* = 67–75) for right-targets, and *M* = 71.56 (*Range* = 66–74) for redundant targets. Collapsed across target-present and target-absent trials, the mean accuracy rate was very high, *M* = 0.97 (*Range* = 0.93–greater than 0.99).

#### RTs

In all experiments, RTs were only analyzed for correct target-present trials (i.e., excluding false-positive responses). Inspection of the data suggested that participants generally complied with the instructions to respond to targets bimanually, making button presses with both thumbs in quick succession. Analyses were carried out using the RT for the faster of the two button presses for each trial.

**Fig. 3:**
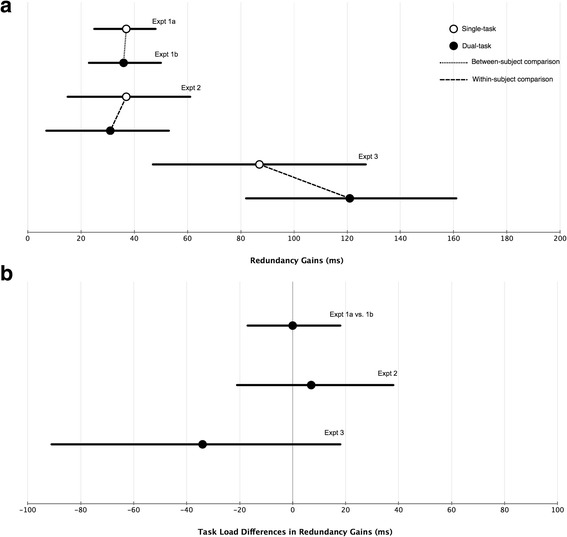
**a** Means and 95% BCIs for redundancy gains (ms) by experiment. **b** Means and 95% BCIs for task-load differences in redundancy gains (single-task RT minus dual-task RT) by experiment

Data showed no credible difference in RTs between single targets on the left (*M* = 577 ms, 95% Bayesian Credible Interval (BCI) (Kruschke, 2015) = [536, 618]) and on the right (*M* = 568 ms, 95% BCI = [527, 610]), (left-right difference: *M*_*Diff*_ = 9 ms, 95% BCI = [−4, 22], *d* = 0.24). The mean single-target RT provides a measure of baseline response speed independent of any redundancy gain. Collapsed across the two single-target conditions, the mean single-target RT was 573 ms, 95% BCI = [532, 613]. Figure 2 shows the 95% BCIs for the mean single-target RT, as well as corresponding task-load differences, for Experiment 1a and the following experiments. As noted above, redundancy gains were calculated by subtracting RT for the redundant-target condition from RT for the faster single-target condition (left or right) for each participant. Even by this conservative measure, data gave clear evidence of a redundancy gain (redundant signals effect: *M*_*RSE*_ = 37 ms, 95% BCI = [25, 48], *d* = 1.86), with the redundant-target condition (*M* = 523 ms, 95% BCI = [482, 564]) producing shorter RTs than the faster single-target condition (*M* = 560 ms, 95% BCI = [519, 601]). Figure 3 presents the redundancy gain and task-load difference for each experiment.

#### Resilience

**Fig. 4:**
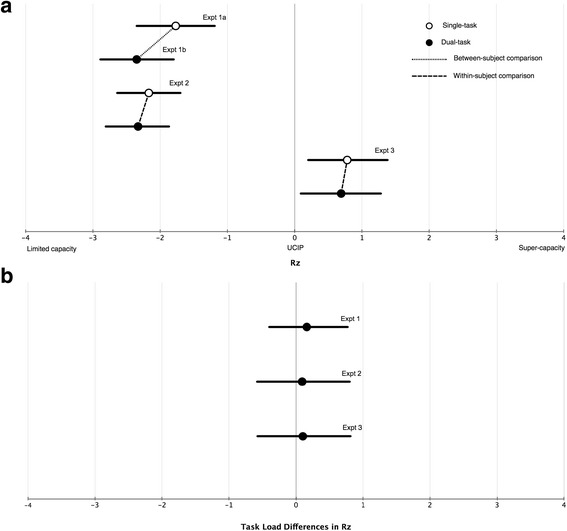
**a** Means and 95% BCIs for standardized resilience scores in Experiments 1 to 3. **b** Means and 95% BCIs for task-load differences in *Rz* (single-task minus dual-task) across experiments

As noted above, the standardized score *Rz* represents the normalized mean of *R*(*t*) across values of *t*, weighted inversely by the variability of *R*(*t*) at each time point (Houpt & Townsend, 2012). Values equivalent to zero represent UCIP processing, positive values indicate super-capacity, and negative values represent limited capacity. Mean *Rz* was credibly negative (*M*_*Rz*_ = − 1.77, 95% BCI = [−2.35, − 1.19]). Figure 4 compares resilience, and task-load differences in resilience, by experiment.

#### Tracking performance

In Experiment 1a, the participant-controlled cursor was invisible, and participants were told to ignore the movements of the red dot of the tracking task. However, joystick movements were recorded. These data provided an estimate of chance-level tracking accuracy, suitable as a baseline against which to compare active tracking performance in the subsequent dual-task experiments. Performance was measured by calculating the RMSE in angular distance of the cursor position relative to the target position. Mean RMSE was 31.34°, 95% BCI = [26.01, 36.66]. If participants followed instructions to ignore the tracking task in Experiment 1a and perform it in the subsequent dual-task experiments, RMSE should be smaller in the later experiments.

### Discussion

The goal of Experiment 1a was to provide a baseline measure of processing efficiency before any secondary task load was added. Resilience for redundant-target processing was highly limited, despite attention being wholly focused on the target detection task. Thus, within a standard distractor-present redundant-target task, the RT gains produced by redundant target presentation were smaller than predicted by statistical facilitation in a UCIP model.

## Experiment 1b

Experiment 1b replicated the procedure of Experiment 1a but with the addition of a central manual tracking task, to test whether concurrent task load reduced processing resilience.

### Method

#### Participants

As we aimed to match sample size from Experiment 1a, we ran participants until we had data for 25 participants who met the inclusion criteria for detection error rates. We achieved this goal after running 29 participants (see “Error rates” section below for details on reasons for exclusions). All participants were undergraduate students (18 female, *M*_*Age*_ = 23.00 years, *SD* = 8.86, *Range* = 18–55), who received either AU$10 or course credit for their participation. None had participated in the previous experiment. All were right-hand dominant (*M*_*FLANDERS*_ = 9.24, *SD* = 1.43), fluent in English, and had normal color vision and normal or corrected-to-normal visual acuity. Twenty-three participants held current valid driver’s licenses, with driving experience ranging from 2 to 38 years (*M* = 6.19, *SD* = 8.68).

#### Apparatus and stimuli

The apparatus and stimuli were identical to those used in Experiment 1a, except that the cursor in the pursuit tracking task was made visible.

#### Procedure

In Experiment 1b, participants performed the peripheral target detection and manual tracking tasks concurrently. Participants were encouraged to maintain accuracy on both tasks, while also aiming to minimize RTs on the detection task. As in Experiment 1a, the task involved one block of tracking intervals, comprising one 60-s practice interval followed by 20 60-s experimental intervals. Lastly, participants completed the FLANDERS questionnaire, recorded their driving experience, and were debriefed.

#### Analysis

Analysis was identical to that of Experiment 1a.

### Results

#### Error rates

As with Experiment 1a, participants with false alarm or miss rates greater than 0.30 in any of the three target conditions were excluded from analysis. Data for three participants with excessive false alarm rates (ranging 0.30–0.67) and for one participant with excessive miss rates (as high as 0.79 in the right-single target condition) were excluded. Mean false alarm rates for the remaining 25 participants were much lower (*M* = 0.09, *Range* = 0.01–0.22). Miss rates for the remaining participants were also very low (left single targets: *M* = 0.01, *Range* = 0.00–0.07; right single targets: *M* = 0.01, *Range* = 0.00–0.08; and redundant targets: *M* = 0.01, *Range* = 0.00–0.06). On average, participants correctly responded to approximately the same number of left-target trials (*M* = 70.88, *Range* = 65–74), right-target trials (*M* = 70.84, *Range* = 65–74), and redundant trials (*M* = 71.56, *Range* = 68–74) throughout the testing session. Collapsed across all trials, mean accuracy was very high (*M* = 0.97, *Range* = 0.93–greater than 0.99).

#### RTs

Unlike Experiment 1a, RTs to left-targets (*M* = 618 ms, 95% BCI = [583, 654]) were credibly shorter than those to right-targets (*M* = 638 ms, 95% BCI = [602, 673]), (left-right difference: *M*_*Diff*_ = − 19 ms, 95% BCI = [−36, − 3], *d* = 0.42). The addition of the manual tracking task in Experiment 1b produced a mean single-target RT (*M* = 628 ms, 95% BCI = [593, 663]) marginally longer than that of Experiment 1a, with the BCI on the difference between experiments just excluding 0, (single- minus dual-task difference: *M*_*Diff*_ = − 55 ms, 95% BCI = [−108, − 2], *d* = 0.57). Analysis confirmed that responses in the redundant-target condition (*M* = 574 ms, 95% BCI = [538, 610]) were faster than in the fastest single-target condition (*M* = 610 ms, 95% BCI = [575, 646]), (*M*_*RSE*_ = 36 ms, 95% BCI = [23, 50], *d* = 0.78). However, there was no credible difference between the size of the redundancy gain in Experiment 1b and that in Experiment 1a, (single- minus dual-task difference: *M*_*Diff*_ = 0 ms, 95% BCI = [−17, 18], *d* = 0.04).

#### Resilience scores

Resilience was again limited (*M*_*Rz*_ = − 2.35, 95% BCI = [−2.89, − 1.80]), indicating that redundancy gains were smaller than predicted by a UCIP model. A comparison between *Rz* scores in Experiments 1a and 1b found no credible difference, (single- minus dual-task difference: *M*_*Diff*_ = 0.58, 95% BCI = [−0.21, 1.37], *d* = 0.43).

#### Tracking performance

Mean RMSE was 15.16° (95% BCI = [13.04, 17.25]), credibly lower than in Experiment 1a, (single- minus dual-task difference: *M*_*Diff*_ = 16.18, 95% BCI = [10.47, 21.87], *d* = 1.79). Thus, data suggest that participants in Experiment 1b engaged in the tracking task as instructed.

To test the possibility of a tradeoff in performance between the target detection and tracking tasks, bivariate correlations were calculated between *Rz* scores and RMSE. The credible interval on the correlation included a value of 0.0 but was wide, *r*(23) = − .14, 95% BCI = [− .58, .30], indicating that the data lacked resolution to strongly support or discredit the possibility of tradeoffs between the two tasks.

### Discussion

Experiments 1a and 1b tested whether a manual tracking task impairs processing efficiency for redundant visual targets. Consistent with previous findings (Eidels et al., 2014; Townsend & Nozawa, 1995), resilience was limited capacity. More surprisingly, resilience did not appear to suffer with the addition of a concurrent, central tracking task.

## Experiment 2

Experiments 1a and 1b failed to find a clear difference in processing efficiency for redundant visual targets between single- and dual-task conditions. However, it is possible that the between-participants design of Experiment 1 simply was not sensitive enough to detect differences between the single- and dual-task conditions. To address this issue, Experiment 2 used a within-participants design to replicate Experiments 1a and 1b, providing a second test of the relationship between task load and target processing efficiency.

### Method

#### Participants

Thirty-two Flinders University students (*M*_*Age*_ = 23.56 years, *SD* = 5.96, *Range* = 18–39) participated for AU$10. No participants had performed any of the previous experiments. Participants were all fluent in English, with normal color vision, and normal or corrected-to-normal visual acuity. In addition, all participants were right-hand dominant (*M*_*FLANDERS*_ = 9.56, *SD* = 0.23), and 28 had current valid driver’s licenses, with experience ranging from 1 to 20 years (*M* = 5.24, *SD* = 5.00).

#### Apparatus and stimuli

Apparatus and stimuli were the same as in Experiment 1.

#### Procedure

The tracking and discrimination tasks were performed in the same way as in Experiment 1. However, each participant completed two blocks of trials. In one block, the participant performed the target-detection task alone, following the same procedure as Experiment 1a (single-task condition). In the other block, the participant performed both tasks simultaneously, as in Experiment 1b (dual-task condition). Block order was counterbalanced across participants. At the beginning of each block, participants were given a 60-s practice session, before completing 20 60-s intervals. Participants were given a short break between blocks. As in the previous experiments, participants finished the testing session by completing the FLANDERS questionnaire and recording their driving experience. The entire session took approximately 50 min.

#### Analysis

Analysis was as in Experiment 1, but was adapted to account for the within-participant manipulation of task load. Analysis of RTs now included additive effects of task load and the interaction of target condition by load (Kruschke, 2015),

$$ {\displaystyle \begin{array}{l}{Y}_{\mathrm{participant},\mathrm{task}\ \mathrm{load},\mathrm{condition}}\sim N\left(a0+{a}_{\mathrm{participant}}+{a}_{\mathrm{task}\ \mathrm{load}}+{a}_{\mathrm{condition}}+{a_{\mathrm{task}\ \mathrm{load}\kern0.5em \times \kern0.5em }}_{\mathrm{condition}},{\sigma_{\mathrm{y}}}^2\right)\\ {}{a}_{\mathrm{participant}}\sim N\left(0,{\sigma_{\mathrm{participant}}}^2\right)\\ {}{a}_{\mathrm{task}\ \mathrm{load}}\sim N\left(0,{\sigma_{\mathrm{task}\ \mathrm{load}}}^2\right)\\ {}{a}_{\mathrm{condition}}\sim N\left(0,{\sigma_{\mathrm{condition}}}^2\right)\\ {}{a_{\mathrm{task}\ \mathrm{load}\kern0.5em \times \kern0.5em }}_{\mathrm{condition}}\sim N\left(0,{{\sigma_{\mathrm{task}\ \mathrm{load}\kern0.5em \times}}_{\kern0.5em \mathrm{condition}}}^2\right)\\ {}{\sigma}_{\mathrm{participant},}{\sigma}_{\mathrm{task}\ \mathrm{load}},{\sigma}_{\mathrm{condition}},{\sigma_{\mathrm{task}\ \mathrm{load}\kern0.5em \times \kern0.5em }}_{\mathrm{condition}}\sim \varGamma\ \left(\alpha, \beta \right)\\ {}\alpha = SD/2\\ {}\beta ={2}^{\ast } SD,\end{array}} $$

where deflections from the grand mean representing the effects of task load, condition, and their interaction were constrained to sum to zero across cells of the design. Likewise, analysis of *Rz* and RMSE included task load as an effect,

$$ {\displaystyle \begin{array}{l}{Y}_{\mathrm{participant}},{\kern0.5em }_{\mathrm{task}\ \mathrm{load}}\sim N\left(a0+{a}_{\mathrm{participant}}+{a}_{\mathrm{task}\ \mathrm{load}},\kern0.5em {\sigma_{\mathrm{y}}}^2\right)\\ {}{\sigma}_{\mathrm{y}}\sim U\left( SD/1000,\kern0.5em {SD}^{\ast }1000\right)\\ {}a0\sim N\left(M,\kern0.5em {\left[100\kern0.5em \times \kern0.5em \mathrm{S}D\right]}^2\right)\\ {}{a}_{\mathrm{participant}}\sim N\left(0,\kern0.5em {\sigma_{\mathrm{participant}}}^2\right)\\ {}{a}_{\mathrm{task}\ \mathrm{load}}\sim N\left(0,\kern0.5em {\sigma_{\mathrm{task}\ \mathrm{load}}}^2\right)\\ {}{\sigma}_{\mathrm{participant}},\kern0.5em {\sigma}_{\mathrm{task}\ \mathrm{load}}\sim \varGamma\ \left(\alpha, \kern0.5em \beta \right)\\ {}\alpha = SD/2\\ {}\beta ={2}^{\ast } SD,\end{array}} $$

where deflections from the grand mean reflecting the effects of task load were constrained to sum to zero across conditions.

### Results

Preliminary inspection found no effect of block order on any of the measures. As such, all analyses were carried out collapsed across block order. Analyses excluded data from three participants with excessive error rates (ranging from 0.44 to 0.89 in any of the target conditions), one participant who appeared not to perform the tracking task in the dual-task condition (32.05° vs. 48.55° RMSE for the single- and dual-task, respectively), and one participant who failed to make enough button-press responses to be analyzed. These exclusions left data from 27 participants for analysis.

#### Error rates

False alarm rates were reasonable in both the single- (*M* = 0.08, *Range* = 0.01–0.23) and the dual-task conditions (*M* = 0.08, *Range* = 0.01–0.21). Miss rates were low for each trial type in the single-task condition (left single: *M* < 0.01, *Range* = 0.00–0.06; right single: *M* < 0.01, *Range* = 0.00–0.03; redundant: *M* < 0.01, *Range* = 0.00–0.03) and in the dual-task condition (left single: *M* = 0.02, *Range* = 0.00–0.17; right single: *M* = 0.01, *Range* = 0.00–0.07; redundant: *M* = 0.01, *Range* = 0.00–0.08). The number of targets correctly detected in each of the three trial conditions was consistent across both the single- (left-targets: *M* = 71.41, *Range* = 67–75; right-targets: *M* = 71.62, *Range* = 69–75; redundant targets: *M* = 71.48, *Range* = 68–75) and dual-task conditions (left-targets: *M* = 70.25, *Range* = 59–74; right-targets: *M* = 71.33, *Range* = 66–75; redundant targets: *M* = 70.71, *Range* = 67–74). Collapsed across target-present and -absent trials, mean accuracy rate was extremely high within both the single- (*M* = 0.98, *Range* = 0.94–1.00) and dual-task conditions (*M* = 0.97, *Range* = 0.89–0.99).

#### RTs

Consistent with Experiment 1a, data showed no difference in mean RT for single targets presented on the left compared with single targets on the right for the single-task block (left: *M* = 601 ms, 95% BCI = [564, 639]; right: *M* = 601 ms, 95% BCI = [563, 637]; *M*_*Diff*_ = 0.79 ms, 95% BCI = [– 22, 25], *d* = 0.08), and in contrast with the results of Experiment 1b, showed no difference in RTs for left vs. right single targets in the dual-task block (left: *M* = 628 ms, 95% BCI = [591, 665]; right: *M* = 633 ms, 95% BCI = [596, 670]; *M*_*Diff*_ = – 5 ms, 95% BCI = [– 29, 18], *d* = 0.14). Mean single-target RT was credibly longer when the tracking task was performed concurrently (*M* = 630 ms, 95% BCI = [595, 665]) than when only the target-detection task was performed (*M* = 601 ms, 95% BCI = [566, 636]), (*M*_*Diff*_ = – 29 ms, 95% BCI = [– 47, – 12], *d* = 0.42). Comparing the fastest single-target RTs (single-task: *M* = 587 ms, 95% BCI = [550, 624]; dual-task: *M* = 612 ms, 95% BCI = [575, 649]) with the redundant RTs (single-task: *M* = 550 ms, 95% BCI = [513, 587]; dual-task: *M* = 581 ms, 95% BCI = [544, 618]) revealed clear redundancy gains of roughly the same size in both the single- (*M*_*RSE*_ = 37 ms, 95% BCI = [15, 61], *d* = 1.47) and dual-task (*M*_*RSE*_ = 31 ms, 95% BCI = [7, 53], *d* = 0.90) conditions, (*M*_*Diff*_ = 7 ms, 95% BCI = [– 21, 38], *d* = 0.26).

#### Resilience

As in the previous experiments, normalized resilience scores for both the single- (*M*_*Rz*_ = – 2.17, 95% BCI [– 2.64, – 1.70]) and dual-task (*M*_*Rz*_ = – 2.33, 95% BCI [– 2.81, – 1.87]) conditions were limited, well below the predictions of the UCIP model (see Fig. 4). Furthermore, comparisons of resilience between the dual- and single-task conditions again failed to find evidence of a difference (*M*_*Diff*_ = 0.16, 95% BCI = [– 0.39, 0.76], *d* = 0.13). These results replicate the findings of Experiment 1, showing no credible effect of task load on dual-channel processing efficiency.

#### Tracking performance

RMSE was substantially smaller in the dual-task block (*M* = 16.87°, 95% BCI = [12.94, 20.78]) than in the single-task block (*M* = 32.97°, 95% BCI = [29.04, 36.86]) (*M*_*Diff*_ = 16.11, 95% BCI = [10.68, 21.46], *d* = 1.07), indicating that participants followed instructions to perform both tasks simultaneously during the dual-task block. Data from the dual-task condition found no evidence of a tradeoff between RMSE and *Rz*, with higher *Rz* scores predicting smaller tracking error, *r*(25) = – .36, 95% BCI = [– .75, .04], although the BCI on this effect included 0.

### Discussion

As in Experiments 1a and b, resilience was highly limited, but was not credibly smaller when participants performed a concurrent manual tracking task. Thus, the tracking and detection tasks did not appear to compete for processing resources (Wickens, 1981, 2002), producing no performance tradeoff between the tasks.

## Experiment 3

The previous experiments found that processing efficiency for redundant visual targets, as measured by resilience, was similar across single- and dual-task conditions. In both cases, resilience was limited, producing mean *Rz* scores decisively below 0. Experiment 3 sought to generalize the results of Experiments 1 and 2 by testing the effects of dual-task load on *Rz* under conditions in which the baseline, single-task resilience scores were not highly limited.

Although neither of the first two experiments included a manipulation to diagnose system architecture, the observed resilience scores suggest that the left and right channels in the target detection task were processed in parallel. As noted above, a serial processing architecture can produce limited-resilience processing. This type of processing only occurs when the time needed to process a distractor is significantly lower than the time needed to process a target (Little et al., 2015). There is little reason to expect that this would have been the case in Experiments 1 and 2. Moreover, past work has shown that TL stimuli can be processed in parallel (Sung, 2008; Yamani, McCarley, Mounts, & Kramer, 2013), at least under conditions in which they are above the limits of sensory resolution and are not subject to visual crowding (Bouma, 1970) or attentional suppression (Yamani et al., 2013).

Experiment 3 measured resilience under single- and dual-task conditions using target and distractor stimuli designed to force serial processing and push resilience above the levels observed in the first two experiments. Targets and distractors were presented in a 4-point font, and embedded in flanking characters intended to produce visual crowding (Bouma, 1970; Whitney & Levi, 2012) in the extrafoveal retina. This design meant that stimulus onsets could still be detected in the retinal periphery. However, to identify targets and distractors, participants had to foveate the stimuli, with little peripheral information to guide participants preferentially toward the target on single-target trials. Assuming that target and distractors required roughly the same amount of time to process, resilience should have reached super-capacity levels (Little et al., 2015), allowing us to test the generality of our findings from the first two experiments.

### Method

#### Participants

We planned for a sample size of 30 participants who met the performance criteria for both the target detection and tracking tasks. To achieve this sample size, 34 undergraduate students from Flinders University (25 female; *M*_*Age*_ = 21.32, *SD* = 3.88, *Range* = 17–35) participated in the experiment either for course credit or for AU$10. No participants had participated in any of the previous experiments. Participants all exhibited normal or corrected-to-normal visual acuity, normal color vision, and English fluency. Participants were required to be right-hand dominant (*M*_*FLANDERS*_ = 9.48, *SD* = 1.93); one participant who failed this requirement was immediately excluded from the study prior to further analysis. Thirty-one participants reported holding a current valid driver’s license, and the mean years driving experience was 3.83 years (*SD* = 3.19; *Range* = 1–15).

#### Apparatus and stimuli

The apparatus was the same as above. Stimuli were similar except for the following changes. First, stimuli for the target detection task were reduced to 4-point font, with each letter subtending approximately 0.44° × 0.35° of visual angle. Second, targets and distractors were embedded within five-item letter arrays. Target letters appeared in the same upper left and upper right locations as in Experiments 1–2, but were flanked on both sides by two letters randomly and independently selected from the set F, H, K, M, N, V, W, X, and Z. To avoid overlap between letters, target and distractor orientations were fixed at 0°. For an illustration of a dual-task single-target trial from Experiment 3, please return to Fig. 1b.

#### Procedure and analysis

Procedure and data analysis were identical to Experiment 2.

### Results

Data from one participant were removed from analysis for high false alarm rates in both the single- (0.53) and dual-task (0.61) conditions. Furthermore, data from two participants who produced roughly equal tracking error in both the single- and dual-task conditions (e.g., 27.58° vs. 29.64°, respectively) were also excluded.

#### Error rates

False alarm rates for the remaining 30 participants were acceptable within both the single- (*M* = 0.07, *Range* = 0.00–0.19) and dual-task (*M* = 0.08, *Range* = 0.00–0.26) conditions. Similarly, target miss rates were low in all trial types for both the single-task condition (left single: *M* = 0.01, *Range* = 0.00–0.16; right single: *M* = 0.02, *Range* = 0.00–0.20; redundant target: *M* = 0.01, *Range* = 0.00–0.18) and the dual-task condition (left single: *M* = 0.03, *Range* = 0.00–0.15; right single: *M* = 0.03, *Range* = 0.00–0.14; redundant target: *M* = 0.02, *Range* = 0.00–0.14). Collapsed across target-present and target-absent trials, mean accuracy rate was high and approximately equal in both the single (*M* = 0.97, *Range* = 0.84–greater than 0.99) and dual-task (*M* = 0.96, *Range* = 0.87–greater than 0.99) conditions. A similar number of targets were detected within each of the three trial types for both the single- (left single: *M* = 70.60, *Range* = 61–74; right single: *M* = 70.33, *Range* = 59–74; redundant target: *M* = 70.90, *Range* = 59–74) and dual-task conditions (left single: *M* = 69.77, *Range* = 58–74; right single: *M* = 69.70, *Range* = 60–73; redundant target: *M* = 70.13, *Range* = 62–73).

#### RTs

Comparisons of RTs for left and right single targets revealed faster responses for targets on the left than those on the right for both the single- (left: *M* = 794 ms, 95% BCI = [749, 838]; right: *M* = 976 ms, 95% BCI = [932, 1019]; *M*_*Diff*_ = − 182 ms, 95% BCI = [−227, − 139], *d* = 1.11) and dual-task conditions (left: *M* = 880 ms, 95% BCI = [836, 924]; right: *M* = 1031 ms, 95% BCI = [987, 1074]; *M*_*Diff*_ = − 151 ms, 95% BCI = [−194, − 105], *d* = 1.43), indicating that participants adopted a left-to-right scanning strategy under serial processing conditions. Mean single-target RT was credibly faster in the single-task condition (*M* = 885 ms, 95% BCI = [847, 923]) than the dual-task condition (*M* = 955 ms, 95% BCI = [917, 994]), (*M*_*Diff*_ = − 70 ms, 95% BCI = [−101, − 40], *d* = 0.50). We found clear evidence for redundancy gains when comparing the fastest single-target RTs (single-task: *M* = 776 ms, 95% BCI = [733, 818]; dual-task: *M* = 877 ms, 95% BCI = [835, 920]) with redundant-target RTs (single-task: *M* = 688 ms, 95% BCI = [646, 730]; dual-task: *M* = 756 ms, 95% BCI = [714, 798]) for both levels of task load (single-task: *M*_*RSE*_ = 87 ms, 95% BCI = [47, 127], *d* = 1.41; dual-task: *M*_*RSE*_ = 121 ms, 95% BCI = [82, 161], *d* = 2.21). Although there was a trend for larger redundancy gains in the dual-task condition, the BCI on the difference score contained 0 (*M*_*Diff*_ = − 34, 95% BCI = [−91, 18], *d* = 0.50).

#### Resilience

As expected, and in contrast to the results of the first three experiments, normalized resilience scores for both the single- (*M*_*Rz*_ = 0.78, 95% BCI = [0.20, 1.38]) and dual-task conditions (*M*_*Rz*_ = 0.69, 95% BCI = [0.09, 1.28]) were credibly super-capacity. However, consistent with the previous experiments, task load did not influence processing resilience (single- minus dual-task difference: *M*_*Diff*_ = 0.10, 95% BCI = [−0.57, 0.81], *d* = 0.06).

#### Tracking performance

RMSE was decisively smaller in the dual-task condition (*M* = 15.69°, 95% BCI = [11.91, 19.49]) than the single-task condition (*M* = 33.11°, 95% BCI = [29.29, 36.87]), (*M*_*Diff*_ = 17.41, 95% BCI = [12.47, 22.28], *d* = 1.39). As in Experiment 2, data trended in the opposite direction to a tradeoff between RMSE and *Rz* scores, *r*(28) = − .35, 95% BCI = [− .72, .02], with the credible interval just overlapping 0.

### Discussion

Experiment 3 assessed target processing efficiency within a forced serial process paradigm. As expected, serial scanning produced super-capacity processing of redundant targets (Little et al., 2015). Notably, the large difference in resilience scores found in the current experiment versus those in the earlier experiments supports the idea that, when stimuli were above sensory thresholds and not compromised by crowding, target processing was in parallel and with limited-capacity. But, despite the difference in processing architecture between experiments, none of the experiments found an effect of task load on processing efficiency. Resilience remained largely unaffected by variations in task load, despite large variations in baseline resilience values and changes to processing architecture.

## General discussion

The current studies examined redundant-target processing within a dual-task paradigm. As expected, a concurrent manual tracking task increased RTs for target detection in the periphery. But, despite this difference in baseline target detection times, the efficiency with which redundant targets were processed was invariant with task load. In other words, a central task slowed responses to peripheral targets, but did not change the rate at which multiple targets were processed relative to single targets. This effect was true regardless of whether targets were processed in parallel with limited resilience (Experiments 1–2), or in serial with super-capacity resilience (Experiment 3).

One interpretation is that the central manual tracking task and the peripheral target detection task tapped into partially independent pools of information-processing resources (Wickens, 2002). Although multiple resource theory includes visual attention as one form of processing resource, it posits separate pools of processing resources for both focal and ambient vision, linking focal processing to the central visual field and ambient to the peripheral visual field. The theory thus allows that the task-load manipulation might not have affected processing efficiency because the tracking task engaged central resources and the target detection task engaged ambient resources. Contrary to this hypothesis, though, mean single-target RTs for dual-task conditions were credibly longer than those for single-task conditions in across all experiments. These results suggest the central tracking and peripheral detection tasks likely tapped common processing resources, presumably at the stages that Wickens (2002) labels perception or cognition; the target and distractor stimuli of Experiment 3 were in fact designed to be indiscriminable in ambient vision, ensuring competition for focal attention between the central and peripheral tasks. Moreover, the Wickens (2002) model proposes that focal processes are specialized for detailed object perception and recognition, whereas ambient processes are specialized for spatial processing. Assuming that participants fixated near the display center to perform the tracking task, the central and peripheral processing demands in the current experiments, therefore, would not have aligned well with the attentional pools hypothesized by multiple resource theory. To optimize the distribution of resources under the model, participants would have had to fixate near the boundary of the display while tracking the moving target with peripheral vision. Eye movement data might test whether any participants adopted such a strategy, or to estimate more generally how often eye movements occurred between the central and peripheral tasks. At best, though, the data indicate that distributing task load over different resource pools would have attenuated dual-tasks costs, not eliminated them.

An alternative explanation for the present results could be that even when redundant peripheral targets were themselves processed in parallel, attention shifted between the central and peripheral tasks in serial (Wickens & Gopher, 1977). By this account, participants would have performed the central tracking task while using a diffuse attentional window to monitor the display periphery for targets and distractors (Steelman et al., 2013; Van der Stigchel et al., 2009). The visual transients produced by peripheral stimulus onsets would have interrupted the central tracking task (Yantis & Jonides, 1990), drawing attention towards the target and distractor stimuli for identification. In Experiments 1–2, attention in this interval would have been spread broadly over the left and right stimuli, processing them in parallel. By contrast, the design of the stimuli in Experiment 3 would have demanded that attention focus on the stimuli in serial, through a series of saccadic eye movements. In both cases, after detecting a target or confirming that both peripheral items were distractors, attention would have returned to the tracking task. Resilience would have been similar across the single- and dual-task blocks, because, in both cases, attention would have been disengaged from the central task while peripheral items were being processed.

One caveat of this attention-switching account is that such a theory predicts a positive association between tracking error and resilience for the redundant targets, whereas our data trended in the opposite direction. The tendency toward better tracking among participants with higher resilience hints at individual differences in effort or ability, differences that might have masked any tradeoffs between tracking and resilience. To better understand how attention is divided between the two tasks, future experiments might employ eye-tracking to identify participants’ attentional strategies, and to test for evidence of discrete attention shifts between the center and periphery.

In application, our results indicate that redundant visual signals are likely to be as effective at aiding visual detection under multi-task conditions as under single-task conditions. This means both that redundant coding will be useful within multi-task workspaces, and that the results of single-task pilot testing can be used to predict the magnitude of RT gain that redundant signals will purchase in a multi-task environment. Thus, design guidelines for complex visual workspaces, such as pilot cockpits or vehicle dashboards, should encourage the use of redundant coding of visual alerts for enhancing detection.

The data also imply a tradeoff between redundancy gains and display complexity. We find that redundant visual targets in peripheral visual displays are of greatest value for low-salience stimuli, those that demand focused attention for detection or recognition, such as when monitoring a large set of gauges or meters. Stimuli of higher salience, discriminable enough to be processed in parallel, are more likely to be processed with limited resilience and with far more modest redundancy gains. This pattern suggests that as a general guideline, display designers might trade redundant target presentation against target salience, reserving highly salient display modes for the most critical signals and presenting information that is less urgent but still time-sensitive in lower salience, redundant signals. By using redundant presentation as a substitute for high conspicuity, this strategy would reduce the risk of a salience-saturated environment in which high-contrast signals compete for attention or overwhelm the operator.

Notably, our findings only consider identical redundant visual signals. Thus, additional work will be necessary to generalize the results to environments in which redundant signals are non-identical (Ben-David & Algom, 2009) or to environments involving auditory or multimodal stimuli (Diederich & Colonius, 2004; Fox, Glavan, & Houpt, 2014). As many warning technologies and displays employ multimodal signals (Rowe & Halpin, 2013; Selcon et al., 1995), further research should examine whether redundant non-identical or multimodal signals also produce equally efficient processing benefits within single- and multi-task environments.

## Conclusions

Within a peripheral redundant-target paradigm, data give no evidence for poorer target processing efficiency while under the load of a secondary tracking task. As expected (Little et al., 2015), however, data do show variations in processing efficiency as a function of display characteristics. Findings suggest there is a modest benefit to employing redundant targets in peripheral visual displays (e.g., on a vehicle dashboard) for situations in which targets are processed in parallel. However, we find that redundant displays have more substantial benefits for target items that demand serial processing.

## Abbreviations

BCI: Bayesian Credible Interval (Kruschke, 2015)
*C*(*t*): Capacity coefficient (Townsend & Nozawa, 1995)
CDF: Cumulative distribution function
*Cz*: Standardized capacity coefficient (Houpt & Townsend, 2012)
*d*: Cohen’s *d*
FLANDERS: Flinders Handedness Survey (Nicholls, Thomas, Loetscher, & Grimshaw, 2013)
*h*(*t*): Hazard function
*H*(*t*): Integrated hazard function
*M*_*Age*_: Mean age
MCMC: Markov chain Monte Carlo
*M*_*Diff*_: Mean difference between conditions
*M*_*FLANDERS*_: Mean Flinders Handedness Survey score
*M*_*RSE*_: Mean redundant signals effect
*M*_*Rz*_: Mean standardized resilience score
*R*(*t*): Resilience coefficient (Little, Eidels, Fific, & Wang, 2015)
RMSE: Root mean squared error
RSE: Redundant signals effect
RT: Response time
*Rz*: Standardized resilience coefficient (Houpt & Little, 2017)
SST: Serial self-terminating
UCIP: Unlimited capacity independent processing
